# We have ‘a very good chance’ of eliminating blinding trachoma by 2020

**Published:** 2013

**Authors:** Elizabeth Kurylo

**Affiliations:** Communications Manager: International Trachoma Initiative, Decatur, USA. ekurylo@taskforce.org

Former US President Jimmy Carter, speaking at the 15th anniversary of Pfizer's donation of Zithromax® in New York on 5th November 2013, said: “With the help of Pfizer, we are trying to eliminate blinding trachoma from the face of the earth by 2020. I think we have a very good chance of reaching this goal.”

During the past 15 years, Pfizer – through the International Trachoma Initiative (ITI) – has donated more than 340 million doses of Zithromax® to 28 countries in Africa and Asia.

After years of untreated trachoma infection, the eyelids turn inward and the eyelashes scrape the cornea with every blink, causing pain and gradual loss of vision from scarring of the cornea. An estimated 320 million people worldwide are at risk of contracting trachoma.

Mark Rosenberg, Director of the Task Force for Global Health, which includes the ITI programme, said: “Many of those at risk are children and their mothers living in the poorest villages in the world with inadequate clean water and sanitation, but trachoma can be prevented, treated and eliminated.”

Pfizer and the International Coalition for Trachoma Control (ICTC), which includes the Carter Center and ITI, support the Global Alliance for the Elimination of Trachoma by the year 2020 (GET 2020), an initiative led by the World Health Organization (WHO). This international alliance for elimination of blindness from trachoma implements the SAFE strategy, approved by the WHO, to prevent and treat trachoma. SAFE stands for: Surgery for the inturned eyelashes (trichiasis); Antibiotics to treat active infection; promotion of Facial cleanliness; and Environmental improvements, including better water supply and latrines to reduce the spread of disease by flies.

**Figure:**
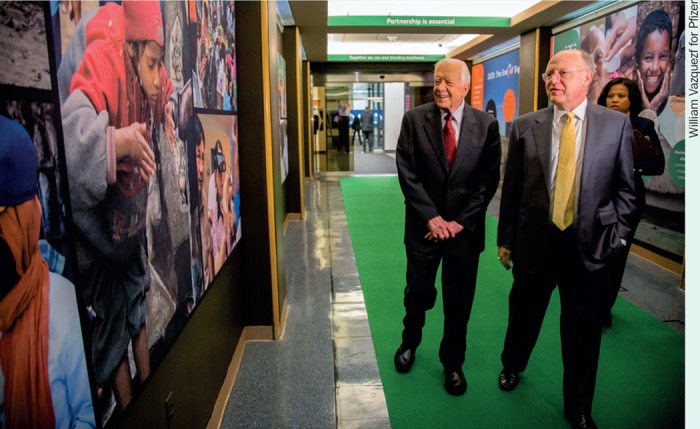
Former President Jimmy Carter (left) with Pfizer CEO Ian Read and Pfizer Senior Director of Social Investments Kimberly Lewis at an event to mark the 15th anniversary of Pfizer's donation of Zithromax(R) to combat trichoma

President Carter said Pfizer's donation of Zithromax® was “momentous in trachoma control,” and added that it allowed the Carter Center and other international nongovernmental organizations to “get the medicine into the villages and demonstrate the world can end blinding trachoma”. He continued: “Millions of people worldwide will be spared the injustice, indignity and pain of their eyelashes scratching and scarring their eyes.”

Pfizer CEO Ian Read said Jimmy Carter's support is key to the success of trachoma elimination, and Carter concluded: “Once people in a village know what needs to be done to get rid of trachoma, they are much more eager than any of us in this room to see it done.”

For 2014, the Trachoma Expert Committee of ITI has approved 63 million doses of Zithromax® for treatment of trachoma in 23 countries.

**Approaching elimination: Mali's post-endemic surveillance strategy**

In Mali, the prevalence of trachoma in 43 out of the originally 51 trachoma endemic districts is low enough so that mass drug administration (MDA) at the district level is no longer warranted. The National Blindness Prevention Programme in Mali began piloting an innovative post-endemic surveillance protocol in 2011 to assess whether high-prevalence pockets of active infection exist within districts that are eligible to stop district-level MDA. The assessment is integrated into surgical camps led by teams of surgeons that come from the capital and travel to areas where trachoma is endemic.

Post-endemic surveillance is conducted in districts where the prevalence of follicular trachoma (TF) is ≤ 10% in children aged 1–9 years.

The surveillance method involves selecting two or four villages in each area in which to do a more detailed investigation. If the population in the area is less than 200,000, two villages are chosen, and if it is between 200,000 and 400,000, four villages are chosen.

In each village, 50 children under the age of 10 are examined for TF.

- If < 5% of children examined have TF, only the children with TF, their families, and surrounding neighbours are treated with antibiotics (azithromax).
- If 5–9.9% of the children have TF, then the entire village is treated.
- If ≥10% of the children have TF, then the entire health district/area is treated.

To date, this post-endemic surveillance strategy has been implemented in 19 districts, and will be scaled up dramatically as Mali approaches the elimination date of 2015. It is seen as a cost-efficient strategy since it integrates surgical camps and active infection surveillance. Having strong and cost-efficient surveillance mechanisms in place to detect areas with a high prevalence of trachoma is critical to reaching full trachoma elimination.

*With thanks to Sanoussi Bamani, Seydou Goita, Yaya Kamissoko, Sadi Moussa, Sidi Coulibaly, Aryc W Mosher, and Emily Toubali*.

